# ﻿High variability in chromosome number from a clade within the gourami family (Teleostei, Osphronemidae)

**DOI:** 10.3897/compcytogen.19.132971

**Published:** 2025-02-25

**Authors:** Brendan Mobley, Andrew P. Anderson

**Affiliations:** 1 Biology Department, Reed College, Portland, Oregon 97202, USA Biology Department, Reed College Portland United States of America

**Keywords:** Fish cytogenetics, gourami, karyotype evolution, 2n reduction

## Abstract

Identifying clades with extensive and conspicuous changes in diploid chromosome number (2n) is an important step in unraveling the evolutionary mechanisms underlying karyotype evolution. Here, we report low 2n in a monophyletic group of teleost fishes within the family Osphronemidae defined by their unique spiral egg structure (the “spiral egg” clade). We sampled seven of the nine known species within the spiral egg clade, reporting novel 2n for five species and confirming two others. We find high variability in both 2n and chromosome arm number (fundamental number, FN), suggesting a 2n reduction during the emergence of the clade and numerous large-scale mutations across evolutionary time. These data provide important information in cataloguing 2n shifts in teleost fishes and highlight this group for further study in chromosomal and genomic evolution due to their karyotypic heterogeneity.

## ﻿Introduction

Chromosome numbers vary widely across vertebrate lineages, but the driving factors behind this variation remain unclear (Damas et al. 2021). Among vertebrates, teleost fishes exhibit some of the most dramatic karyotype evolution patterns, including frequent and rapid changes in diploid chromosome number (2n), high intra-genus chromosome diversity, and notable variability in chromosomal arm number (FN). For example, the genus *Nothobranchius* Peters, 1868 demonstrates extreme 2n variability (2n = 16–50, Krysanov and Demidova 2018), while *Corydoras* Lacepède, 1803 (2n = 40–134, Shimabukuro-Dias et al. 2004; Marburger et al. 2018) and the family Salmonidae (2n = 52–102, Phillips and Ráb 2001) show complex karyotype diversification following polyploidy events. Other groups, such as the family Eleotridae, display a remarkable range in FN (FN = 38–98, da Silva et al. 2021). As a whole, teleosts have the widest 2n range of all vertebrates, from 2n = 12 in the marine species *Sigmopsbathyphilus* Vaillant, 1884 (Post 1974) to 2n = 417–470 in the freshwater species *Ptychobarbusdipogon* Regan, 1905 (Yu and Yu 1990). Despite this diversity, teleost fishes show a strong trend of conserved karyotypes (Galetti et al. 2000; Mank and Avise 2006; Nakatani et al. 2007) with over half of all karyotyped fish species having a diploid number of 48 or 50 (Mank and Avise 2006; Arai 2011). Identifying clades with unusual changes in 2n or FN, such as large reductions or rapid shifts, is particularly valuable for understanding chromosome evolution in teleosts.

An intriguing group of fishes with apparent variance in 2n is found within the family Osphronemidae. Commonly called gouramis, most osphronemids have 2n = 46–48 (Suppl. material 1), constituting a stable 2n pattern. Two notable deviations from this trend are the chocolate gourami, *Sphaerichthysosphromenoides* Canestrini, 1860, which has the lowest recorded diploid chromosome number in the family (2n = 16, Calton and Denton 1974) and the pikehead gourami, *Luciocephaluspulcher* Gray, 1830, which was reported in a personal communication to have 2n = 20 (Arai 2011). These low counts represent a huge 2n reduction compared to the rest of the family. Moreover, such low diploid chromosome numbers are exceedingly rare among fishes (Arai 2011).

**Figure 1.**
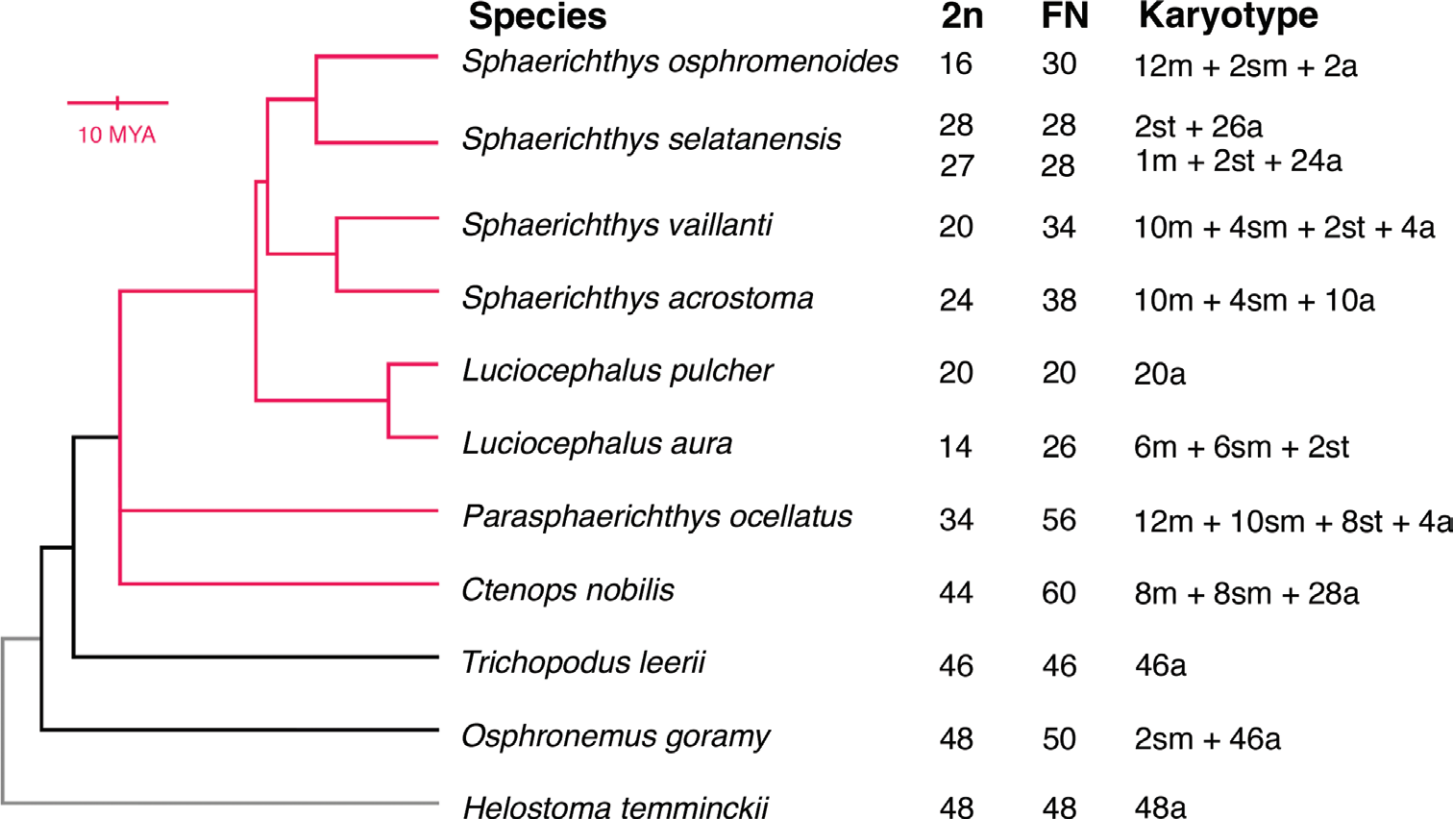
Selected karyotypes from species in the order Anabantiformes. Phylogenetic relationships are from Ruber et al. (2006) and are shown to scale for the spiral egg clade (red) but not the selected species in the family Osphronemidae (black) or the outgroup (Helostomatidae, grey). Diploid chromosome number (2n), chromosomal arm number (FN), and Karyotype for the four species not generated in this study can be found in Arai (2011) and Grazyna et al. (2008). Karyotype describes number of metacentric (m), submetacentric (sm), subtelocentric (st), and acrocentric (a) chromosomes. FN considers metacentric and submetacentric chromosomes as biarmed and subtelocentric and acrocentric chromosomes as uniarmed.

Both *S.osphromenoides* and *L.pulcher* are members of the “spiral egg” clade, a monophyletic group within the family Osphronemidae that includes the genera *Sphaerichthys* Canestrini, 1860, *Luciocephalus* Bleeker, 1850, *Parasphaerichthys* Prashad et Mukerji, 1929, and *Ctenops* McClelland, 1845 (Fig. 1). The monophyly was initially proposed based on the unique morphology of their eggs, which are covered in projections arranged in a spiral pattern, and later confirmed and refined through molecular evidence (Britz et al. 1995; Rüber et al. 2006). Another differentiating feature of the spiral egg clade is an angular jaw shape, which is most pronounced in the highly derived, pike-like morphology of the piscivorous genus *Luciocephalus*. The spiral egg clade is also notable for having the only species (*S.osphromenoides* and *Sphaerichthysselatanensis* Vierke, 1979) in the family Osphronemidae with female broodcare via mouthbrooding, compared to the overwhelmingly male mouthbrooders or bubble nesters in the family (Rüber et al. 2006), although recent evidence has called into question the sex of caring parent in *S.osphromenoides* (Zworykin et al. 2024). Chromosomes of the spiral egg clade remain largely uninvestigated; besides *S.osphromenoides* and *L.pulcher*, only one other species has been studied cytogenetically (*Ctenopsnobilis* McClelland, 1845, 2n = 44: Rishi et al. 1997). Given the low 2n of *S.osphromenoides* and *L.pulcher* and the large 2n reduction relative to the broader family Osphronemidae, we aim to characterize the karyotypes of additional members within the spiral egg clade. This information will allow us to describe the karyotypic diversity and evolutionary history of this remarkable group, thereby contributing an extraordinary example to the 2n diversity observed in teleost fishes.

## ﻿Material and methods

All species (Fig. 1) were sourced from the aquarium trade (Wet Spot Tropical Fish, Portland, Oregon, USA; Nationwide Aquatics, Tinley Park, Illinois, USA; Aqua Imports, Boulder, Colorado, USA), and housed in species-specific tanks (110 liters) on a shared flow-through system. Water parameters were maintained at pH 7.0, GH 30 ppm, and KH 40 ppm, with a 12-hour light/dark cycle supplemented by 30 minutes of dim lighting to simulate dawn and dusk. All species were fed a combination of live *Artemia* sp. nauplii and adult *Enchytraeusbuchholzi* daily and were inspected for any health issues. Specimens were kept at Reed College and approved by the institutional IACUC under the AUP # 01-2018. Specimens were housed for a minimum of one week to acclimate them to laboratory conditions and ensure good health for optimal cell proliferation. Live specimens were photographed with an iPhone 13.

Chromosome preparations were made following Kligerman and Bloom (1977) with the indicated modifications. Specimens were incubated in 0.005% colchicine solution for 6–7 hours, then euthanized by rapid chilling and dissected to remove gill arches. Sex determination was conducted by gross examination of external morphology and gonads, and photographs were taken throughout. Dissected specimens were stored at -80 °C for future molecular analyses. Gill arches were incubated in 0.4% KCl solution for 20–30 minutes, then fixed in two changes of 3:1 ethanol:acetic acid fixative for at least 30 minutes each, followed by an overnight fixation period at 4 °C. Tissue was then homogenized into suspension by mincing in 50% acetic acid and dropped onto slides pre-warmed to 30–40 °C and air-dried. Slides were examined under phase contrast microscopy to assess quality, then aged at room temperature for at least one day and stained for 10 minutes in 10% Giemsa in pH 6.8 phosphate buffer (Gibco™ Gurr Buffer Tablets) and air-dried.

Chromosomes were examined under a Nikon Eclipse Ti-E microscope operated by Nikon NIS Elements AR software and photographed with an oil immersion objective at 100x magnification and green color filtering using a Hamamatsu ORCA-Flash4.0 camera. Digital images were optimized, then homologous chromosomes were paired by size and morphology and arranged by classification using ImageJ v1.52v and Adobe Photoshop 24.3.0. At least 35 complete metaphase spreads were photographed from each specimen, with completeness defined as the highest consistently observed 2n. Chromosomes were classified as metacentric (m), submetacentric (sm), subtelocentric (st), or acrocentric (a) according to their arm ratios (Levan et al. 1964). Chromosomal arm number (Fundamental Number, FN) was calculated by counting metacentric and submetacentric chromosomes as biarmed and subtelocentric and acrocentric chromosomes as uniarmed (Suppl. material 2).

### ﻿Data availability

Raw images of chromosome spreads and finalized karyotype images can be found here: https://github.com/AndersonDrew/Gourami_Chromosome. Contact authors for any additional data/information.

## ﻿Results

**Figure 2.**
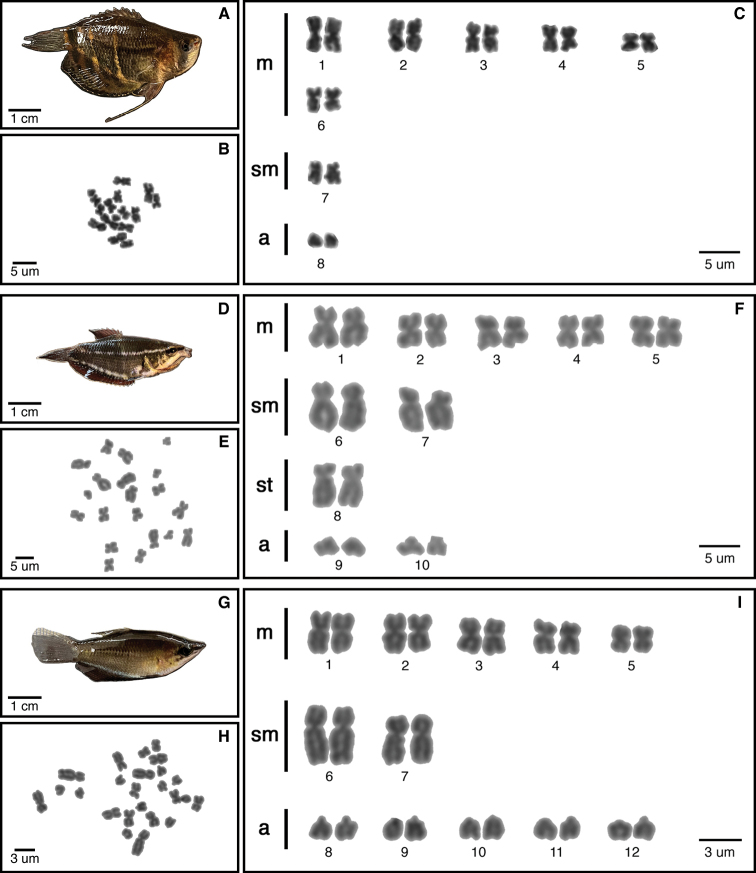
Specimens, metaphase chromosome spreads and karyotypes of *Sphaerichthysosphromenoides* (**A–C**), *S.vaillanti* (**D–F**), *S.acrostoma* (**G–I**) by conventional technique.

**Figure 3.**
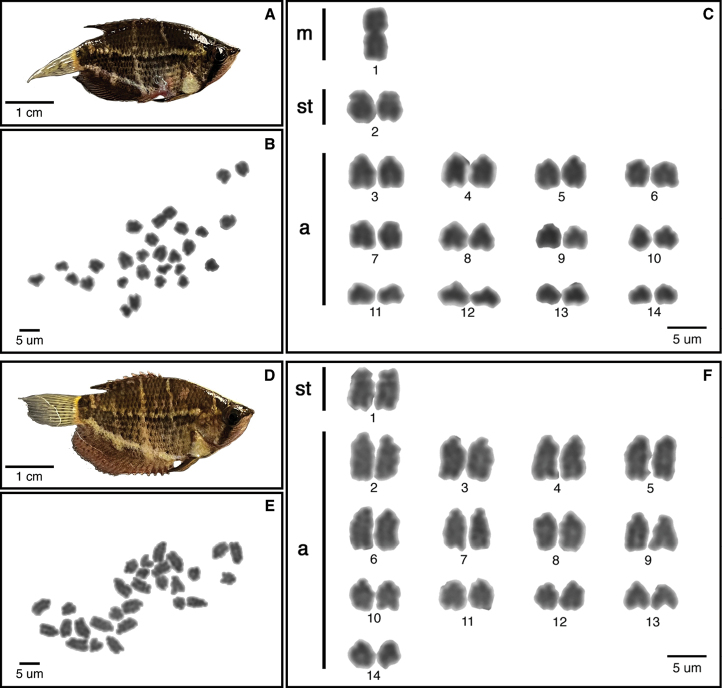
Specimens, metaphase chromosome spreads and karyotypes of *Sphaerichthysselatanensis* with 2n = 27 (**A–C**) and 2n = 28 (**D–F**) by conventional technique.

**Figure 4.**
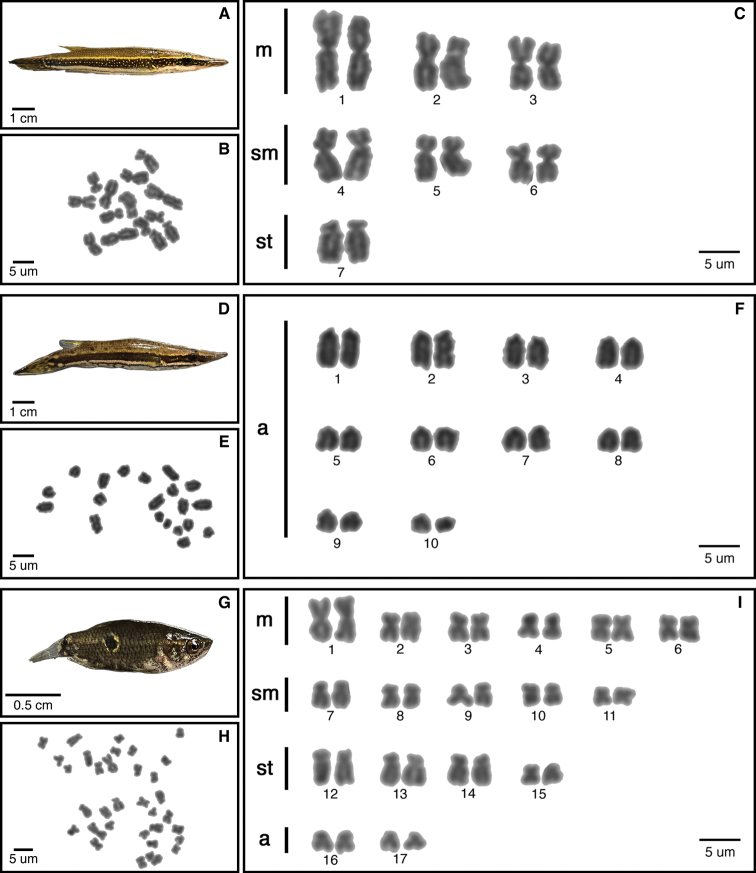
Specimens, metaphase chromosome spreads and karyotypes of *Luciocephalusaura* (**A–C**), *L.pulcher* (**D–F**), *Parasphaerichthysocellatus* (**G–I**) by conventional technique.

We describe karyotypes for the first time in five species (Figs 2–4): *S.selatanensis*, *Sphaerichthysvaillanti* Pellegrin, 1930, *Sphaerichthysacrostoma* Vierke, 1979, *Luciocephalusaura* Tan et Ng, 2005, and *Parasphaerichthysocellatus* Prashad et Mukerji, 1929. We also confirmed the karyotypes of an additional two species (*S.osphromenoides* and *L.pulcher*), which matched those established in the literature (Calton and Denton 1974; Arai 2011). All species in the genus *Sphaerichthys* had distinct 2n values, ranging from 2n = 16–28. The FN varied less, with a range of FN = 28–38. Notably, the sister species *S.osphromenoides* (Fig. 2) and *S.selatanensis* (Fig. 3) had primarily biarmed and primarily uniarmed karyotypes, respectively, resulting in nearly identical FN values despite a 2n difference of 12. The other two sister species in the genus (*S.acrostoma* and *S.vaillanti*) showed a mix of biarmed and uniarmed chromosomes (Fig. 2) and had different values for both 2n and FN. We confirmed that *L.pulcher* had an entirely uniarmed karyotype of 20 acrocentric chromosomes, whereas *L.aura* had a primarily biarmed karyotype with a lower 2n but higher FN than *L.pulcher* (Fig. 4). *P.ocellatus* had a predominantly biarmed karyotype (Fig. 4) with higher 2n and FN values than any *Sphaerichthys* or *Luciocephalus* species, but still lower than those reported for *C.nobilis* in the literature (Arai 2011).

The two *S.selatanensis* individuals we sampled had different karyotypes (Fig. 3). One had 28 uniarmed chromosomes (2n = 28, 2st+26a), while the other had 26 uniarmed chromosomes and a single unpaired metacentric chromosome (2n = 27, 1m+2st+24a). The unpaired metacentric chromosome was approximately twice the size of the largest acrocentric chromosomes and may have arisen from a fused acrocentric pair. Gross examination of the external features and gonads suggested both individuals are male, but without a histological analysis, we caution against ruling out the possibility of a sex-biased chromosomal arrangement.

## ﻿Discussion

We found that the genera *Sphaerichthys*, *Luciocephalus*, and *Parasphaerichthys* have low diploid chromosome numbers (2n≤34) and display high intra-genus variation in both 2n and FN. These trends are not observed in the broader family Osphronemidae, which has relatively stable diploid chromosome numbers around 46–48 (Suppl. material 1), suggesting that the highly variable karyotype evolution began following the divergence of the spiral egg clade about 25 million years ago (Rüber et al. 2006). The large decrease in 2n suggests that the rearrangements were predominantly fusion mutations.

With our current data, we cannot conclusively determine the mechanisms or driving forces behind the high differentiation of these karyotypes. Further study of the spiral egg clade presents an excellent opportunity to understand how these exceptionally differentiated karyotypes arose and could give insight into larger patterns of chromosome evolution. Future work with these species should include histological analysis of the gonads to confirm the gonadal sex with greater accuracy. Notably, most Osphronemidae species have not been examined cytogenetically. Karyotyping more species within the family could uncover additional clades with high karyotype differentiation. Further attention to this cytogenetically diverse group could help resolve outstanding evolutionary questions on chromosomal rearrangements and genome diversity, potentially yielding insights applicable across other vertebrate lineages.

## Supplementary materials

Supplementary material 1 Collection of reported karyotypes across Osphronemidae including our study species Data type xlsx https://binary.pensoft.net/file/1262896This dataset is made available under the Open Database License (http://opendatacommons.org/licenses/odbl/1.0/). The Open Database License (ODbL) is a license agreement intended to allow users to freely share, modify, and use this Dataset while maintaining this same freedom for others, provided that the original source and author(s) are credited.Brendan Mobley, Andrew P. Anderson

Supplementary material 2 Summary statistics of chromosome spreads in this study Data type xlsx https://binary.pensoft.net/file/1262897This dataset is made available under the Open Database License (http://opendatacommons.org/licenses/odbl/1.0/). The Open Database License (ODbL) is a license agreement intended to allow users to freely share, modify, and use this Dataset while maintaining this same freedom for others, provided that the original source and author(s) are credited.Brendan Mobley, Andrew P. Anderson
